# Supplementary figures and images for: A protein microarray analysis of amniotic fluid proteins for the prediction of spontaneous preterm delivery in women with preterm premature rupture of membranes at 23 to 30 weeks of gestation
Source: PLoS One. 2020 Dec 31;15(12):e0244720. doi: 10.1371/journal.pone.0244720 (PMC7774979; doi:10.1371/journal.pone.0244720)

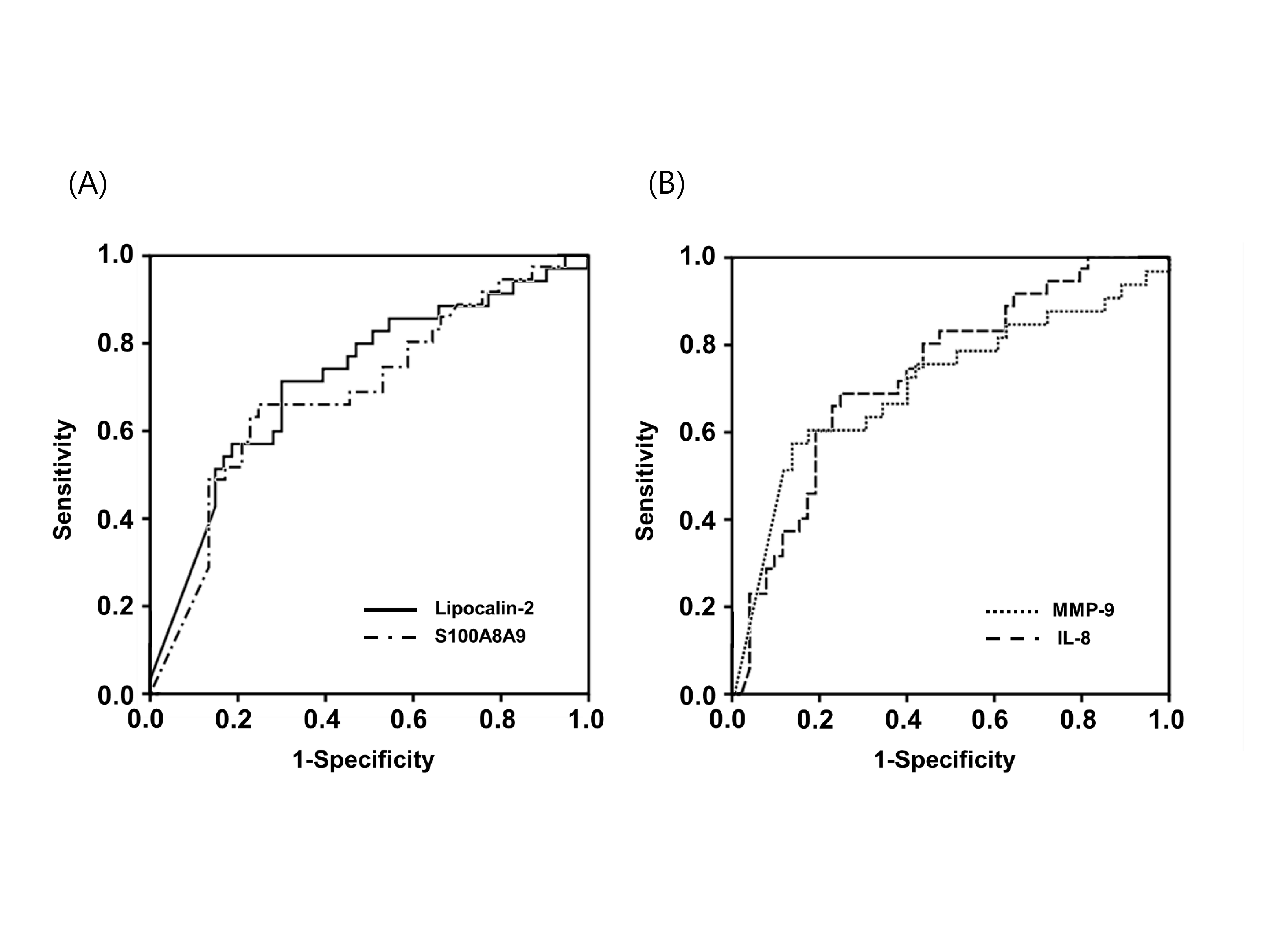

Supplement: S1 Fig — (A) Receiver operating characteristic (ROC) curves of amniotic fluid (AF) lipocalin-2 and S100 A8/A9 at predicting spontaneous preterm delivery (SPTD) within 7 days (AF lipocalin-2: area under the curve [AUC] = 0.717, SE = 0.057; and AF S100 A8/A9: AUC = 0.689, SE = 0.059). (B) ROC curves of AF matrix metalloproteinase-9 (MMP-9) and interleukin-8 (IL-8) at predicting SPTD within 7 days (AF MMP-9: AUC = 0.755, SE = 0.053; and AF IL-8: AUC = 0.737, SE = 0.054). Differences among the AUCs of AF lipocalin-2, S100 A8/A9, MMP-9, and IL-8 were not significant (all variables: P = 0.28–0.73). S100 A8/A9, S100 calcium binding protein A8/A9 complex. (TIF) [file pone.0244720.s001.tif]
