# Supplementary material for: A protein microarray analysis of amniotic fluid proteins for the prediction of spontaneous preterm delivery in women with preterm premature rupture of membranes at 23 to 30 weeks of gestation
Source: PLoS One. 2020 Dec 31;15(12):e0244720. doi: 10.1371/journal.pone.0244720 (PMC7774979; doi:10.1371/journal.pone.0244720)
Supplement: S1 Table — (DOCX) [file pone.0244720.s002.docx]

**S1 Table.** Demographic and clinical characteristics of women with preterm premature rupture of membranes recruited for protein microarray analysis [a case-control study]

|  | Women delivering within 14 days  (n = 15) | Women delivering after 14 days  (n = 15) | *P*-value |
| --- | --- | --- | --- |
| Age (years) | 32.3 ± 3.7 | 31.3 ± 3.8 | 0.641**^a^** |
| Nulliparity | 46.7% (7) | 46.7% (7) | 1.000**^b^** |
| Gestational age at sampling (weeks) | 26.9 ± 1.7 | 26.9 ± 1.6 | 0.992**^a^** |
| Gestational age at delivery (weeks) | 27.4 ± 1.9 | 33.5 ± 3.8 | **<0.001^a^** |
| Use of antibiotics | 86.7% (13) | 100% (15) | 0.483**^b^** |
| Use of tocolytics | 66.7% (10) | 73.3% (11) | 0.690**^b^** |
| Use of corticosteroids | 80.0% (12) | 93.3% (14) | 0.598**^b^** |
| Positive AF cultures | 20.0% (3) | 66.6% (10) | **0.025^b^** |
| Sampling-to-delivery interval (days) | 3.8 ± 3.6 | 45.6 ± 26.5 | **<0.001^a^** |

AF, amniotic fluid.

Data are given as the mean ± SD or % (n).

**^a^ Mann-Whitney *U*-tests.**

**^b^ χ^2^-tests or Fisher’s exact tests,** where **appropriate.**
