# Supplementary material for: A protein microarray analysis of amniotic fluid proteins for the prediction of spontaneous preterm delivery in women with preterm premature rupture of membranes at 23 to 30 weeks of gestation
Source: PLoS One. 2020 Dec 31;15(12):e0244720. doi: 10.1371/journal.pone.0244720 (PMC7774979; doi:10.1371/journal.pone.0244720)
Supplement: S2 Table — (DOCX) [file pone.0244720.s003.docx]

**S2 Table** Characteristics of the study population grouped by spontaneous preterm delivery within 7 days of sampling in the total cohort

| Variables | Spontaneous preterm delivery after sampling | | *P-*value |
| --- | --- | --- | --- |
|  | ≤ 7 days (n = 35) | > 7 days (n = 53) |  |
| Maternal age (years) | 32.2 ± 3.1 | 31.9 ± 3.9 | 0.636**^a^** |
| Nulliparity | 37.1% (13/35) | 47.1% (25/53) | 0.353**^c^** |
| Gestational age at sampling (weeks) | 28.5 ± 1.8 | 27.1± 2.3 | **0.003^b^** |
| Gestational age at delivery (weeks) | 28.9 ± 1.9 | 31.8 ± 3.4 | **< 0.001^b^** |
| Sampling-to-delivery interval (days) | 2.7 ± 2.1 | 32.4 ± 21.9 | **< 0.001^b^** |
| AF endostatin (ng/mL) | 68.6 ± 27.1 | 67.2 ± 23.3 | 0.895**^b^** |
| AF Fas (ng/mL) | 5.2 ± 1.9 | 4.7 ± 1.9 | 0.176**^b^** |
| AF IL-8 (ng/mL) | 9.2 ± 6.5 | 4.2 ± 5.5 | **< 0.001^b^** |
| AF lipocalin-2 (µg/mL) | 1.6 ± 0.9 | 0.8 ± 0.8 | **0.001^b^** |
| AF MMP-9 (ng/mL) | 118.68 ± 93.77 | 37.97 ± 68.28 | **0.001^b^** |
| AF S100 A8/A9 (µg/mL) | 28.6 ± 23.9 | 13.7 ± 19.1 | **0.003^b^** |
| Positive AF cultures | 62.8% (22/35) | 33.9% (18/53) | **0.008^c^** |
| Use of tocolytic agents | 82.8% (29/35) | 62.2% (33/53) | **0.038^c^** |
| Use of antibiotics | 94.2% (33/35) | 96.2% (51/53) | 1.000**^c^** |
| Use of antenatal corticosteroids | 97.1% (34/35) | 84.9% (45/53) | 0.081**^c^** |
| Clinical chorioamnionitis | 11.4% (4/35) | 16.9% (9/53) | 0.552**^c^** |
| Histological chorioamnionitis **^d^** | 74.2% (26/35) | 60.0% (30/50) | 0.172**^c^** |

AF, amniotic fluid; Fas (TNFRSF6), ﬁbroblast-associated **(**tumor necrosis factor receptor superfamily member 6); IL, interleukin; MMP, matrix metalloproteinase; S100A8/A9, S100 calcium binding protein A8/A9 complex.

Data are given as the mean ± standard deviation or % (n/N).

**^a^ Student’s *t*-tests**

**^b^ Mann-Whitney *U*-tests**

**^c^ χ^2^-tests or Fisher’s exact tests,** where **appropriate.**

**^d^** Three cases were excluded for the analysis because delivery took place at another institution.
