# Supplementary material for: A protein microarray analysis of amniotic fluid proteins for the prediction of spontaneous preterm delivery in women with preterm premature rupture of membranes at 23 to 30 weeks of gestation
Source: PLoS One. 2020 Dec 31;15(12):e0244720. doi: 10.1371/journal.pone.0244720 (PMC7774979; doi:10.1371/journal.pone.0244720)
Supplement: S3 Table — Unadjusted and adjusted odds ratios of association between potential amniotic fluid proteins and spontaneous preterm delivery within 7 days in women with preterm premature rupture of membranes in the total cohort. (DOCX) [file pone.0244720.s004.docx]

**S3 Table** Multivariable logistic regression model showing the unadjusted and adjusted odds ratios of association between potential amniotic fluid proteins and spontaneous preterm delivery within 7 days in women with preterm premature rupture of membranes in the total cohort (n = 88)

| Variables | Odds ratio (95% confidence interval) | | |
| --- | --- | --- | --- |
|  | Unadjusted | Adjusted^a^ | *P*-value^b^ |
| AF IL-8 (ng/mL) | 1.140 (1.057 - 1.228) | 1.160 (1.066 – 1.263) | **0.001** |
| AF lipocalin-2 (µg/mL) | 2.356 (1.456 – 3.812) | 2.951 (1.652 – 5.271) | **<0.001** |
| AF MMP-9 (ng/mL) | 1.011 (1.006 – 1.017) | 1.011 (1.005 – 1.018) | **<0.001** |
| AF S100 A8/A9 (µg/mL) | 1.031 (1.010 – 1.053) | 1.032 (1.009 – 1.056) | **0.006** |

AF, amniotic fluid; IL, interleukin; MMP, matrix metalloproteinase; S100A8/A9, S100 calcium binding protein A8/A9 complex.

^a^ For gestational age at sampling and use of tocolytics.

^b^ Of odds ratio adjusted for gestational age at sampling and use of tocolytics.
