# Supplementary material for: A protein microarray analysis of amniotic fluid proteins for the prediction of spontaneous preterm delivery in women with preterm premature rupture of membranes at 23 to 30 weeks of gestation
Source: PLoS One. 2020 Dec 31;15(12):e0244720. doi: 10.1371/journal.pone.0244720 (PMC7774979; doi:10.1371/journal.pone.0244720)
Supplement: S4 Table — (DOCX) [file pone.0244720.s005.docx]

**S4 Table** Diagnostic indices of lipocalin-2, MMP-9, S100 A8/A9, and interleukin-8 in amniotic fluid to predict spontaneous preterm delivery within 7 days of sampling in women with preterm premature rupture of membranes in the total cohort (n = 88)

| Variables | Area (± SE) under the ROC curve**^a^** | 95% CI | Cut-off value^b^ | Sensitivity**^c^**  (95% CI) | Specificity**^c^**  (95% CI) | PPV | NPV |
| --- | --- | --- | --- | --- | --- | --- | --- |
| AF IL-8 (ng/mL) | 0.737 ± 0.054 | 0.631–0.843 | ≥ 5.56 | 68.6 (50.7-83.1) | 75.5 (61.7-86.2) | 64.9 | 78.4 |
| AF lipocalin-2 (µg/mL) | 0.717 ± 0.057 | 0.605-0.829 | ≥ 0.81 | 71.4 (53.6–85.3) | 67.9 (53.6–80.0) | 59.5 | 78.3 |
| AF MMP-9 (ng/mL) | 0.755 ± 0.053 | 0.651-0.859 | ≥ 4.27 | 83.7 (69.3-93.2) | 60.5 (44.4-75.0) | 67.9 | 78.8 |
| AF S100 A8/A9 (µg/mL) | 0.689 ±0.059 | 0.573 – 0.804 | ≥ 11.48 | 65.7 (47.7-80.8) | 75.5 (61.7-86.2) | 63.9 | 76.9 |

SE, standard error; ROC, receiver operating characteristics; CI, confidence interval; PPV, positive predictive value; NPV, negative predictive value; AF, amniotic fluid; IL, interleukin; MMP, matrix metalloproteinase; S100A8/A9, S100 calcium binding protein A8/A9 complex.

**^a^ Receiver operating characteristic curve analysis**

**^b^** Cut-off values corresponding to the highest sum of sensitivity and specificity.

**^c^** Values are presented as % (95% CI).
