# Supplementary material for: A protein microarray analysis of amniotic fluid proteins for the prediction of spontaneous preterm delivery in women with preterm premature rupture of membranes at 23 to 30 weeks of gestation
Source: PLoS One. 2020 Dec 31;15(12):e0244720. doi: 10.1371/journal.pone.0244720 (PMC7774979; doi:10.1371/journal.pone.0244720)
Supplement: S5 Table — (DOCX) [file pone.0244720.s006.docx]

**S5 Table** Characteristics of the study population grouped by preterm delivery at < 34 weeks in the total cohort

| Variables | Preterm delivery at < 34+0 weeks  (n = 71) | Preterm delivery at ≥ 34+0 weeks (n=17) |  |
| --- | --- | --- | --- |
| Maternal age (years) | 32.1 ± 3.6 | 31.7 ±3.9 | 0.716^a^ |
| Nulliparity | 39..4% (28/71) | 58.8% (10/17) | 0.147^c^ |
| Gestational age at sampling (weeks) | 27.6 ± 2.3 | 27.8 ± 2.2 | 0.824^b^ |
| Gestational age at delivery (weeks) | 29.5 ± 2.2 | 35.5 ± 2.2 | **< 0.001**^b^ |
| Sampling-to-delivery interval (days) | 13.3 ± 13.9 | 53.5 ± 22.5 | **<0.001**^b^ |
| AF endostatin (ng/mL) | 68.7 ± 26.0 | 63.9 ± 18.7 | 0.788^b^ |
| AF Fas ( ng/mL) | 5.02 ± 1.94 | 4.40 ± 1.69 | 0.163^b^ |
| AF IL-8 (ng/mL) | 7.2 ± 6.4 | 2.1 ± 4.1 | **<0.001**^b^ |
| AF lipocalin-2 (µg/mL) | 1.27 ± 0.96 | 0.59 ± 0.79 | **0.004**^b^ |
| AF MMP-9 (ng/mL) | 79.74 ± 91.03 | 25.11 ± 57.01 | **<0.001**^b^ |
| AF S100 A8/A9 (µg/mL) | 21.6 ± 22.7 | 11.4 ± 18.9 | **0.023**^b^ |
| Positive AF cultures | 54.9% (39/71) | 5.9% (1/17) | **<0.001**^c^ |
| Use of tocolytic agents | 76.1% (54/71) | 47.1% (8/17) | **0.019**^c^ |
| Use of antibiotics | 97.2% (69/71) | 88.2% (15/17) | 0.167^c^ |
| Use of antenatal corticosteroids | 97.2% (69/71) | 58.8% (10/17) | **<0.001**^c^ |
| Clinical chorioamnionitis | 16.9% (12/71) | 5.9% (1/17) | 0.448^c^ |
| Histological chorioamnionitis ^d^ | 73.2% (52/71) | 28.6% (4/14) | **0.004**^c^ |

AF, amniotic fluid; Fas (TNFRSF6), ﬁbroblast-associated (tumor necrosis factor receptor superfamily member 6); IL, interleukin; MMP, matrix metalloproteinase; S100 A8/A9, S100 calcium binding protein A8/A9 complex.

Data are given as the mean ± standard deviation or % (n/N).

^a^ Student’s *t*-tests

^b^ Mann-Whitney *U*-tests

^c^ χ^2^-tests or Fisher’s exact tests, where appropriate.

^d^ Three cases were excluded for the analysis because delivery took place at another institution.
