# Supplementary material for: A protein microarray analysis of amniotic fluid proteins for the prediction of spontaneous preterm delivery in women with preterm premature rupture of membranes at 23 to 30 weeks of gestation
Source: PLoS One. 2020 Dec 31;15(12):e0244720. doi: 10.1371/journal.pone.0244720 (PMC7774979; doi:10.1371/journal.pone.0244720)
Supplement: S6 Table — Unadjusted and adjusted odds ratios of association between potential amniotic fluid proteins and preterm delivery at < 34 weeks in women with preterm premature rupture of membranes in the total cohort. (DOCX) [file pone.0244720.s007.docx]

**S6 Table.** Multivariable logistic regression model showing the unadjusted and adjusted odds ratios of association between potential amniotic fluid proteins and preterm delivery at < 34 weeks in women with preterm premature rupture of membranes in the total cohort (n = 88)

| Variables | Odds ratio (95% confidence interval) | | |
| --- | --- | --- | --- |
|  | Unadjusted | Adjusted^a^ | *P*-value^b^ |
| AF IL-8 (ng/mL) | 1.255 (1.048 - 1.432) | 1.161 (0.990 – 1.361) | 0.066 |
| AF lipocalin-2 (µg/mL) | 2.567 (1.189 – 5.544) | 2.285 (0.959 – 5.448) | 0.062 |
| AF MMP-9 (ng/mL) | 1.010 (1.001 – 1.019) | 1.009 (0.999 – 1.020) | 0.078 |
| AF S100 A8/A9 (µg/mL) | 1.027 (0.995 – 1.060) | 1.016 (0.982 – 1.052) | 0.354 |

AF, amniotic fluid; IL, interleukin; MMP, matrix metalloproteinase; S100 A8/A9, S100 calcium binding protein A8/A9 complex.

^a^ For use of tocolytics and corticosteroids.

^b^ Of odds ratio adjusted for use of tocolytics and corticosteroids.
