# Supplementary material for: A protein microarray analysis of amniotic fluid proteins for the prediction of spontaneous preterm delivery in women with preterm premature rupture of membranes at 23 to 30 weeks of gestation
Source: PLoS One. 2020 Dec 31;15(12):e0244720. doi: 10.1371/journal.pone.0244720 (PMC7774979; doi:10.1371/journal.pone.0244720)
Supplement: S7 Table — (DOCX) [file pone.0244720.s008.docx]

**S7 Table** Characteristics of the study population grouped by spontaneous preterm delivery within 14 days of sampling in the cohort after excluding the patients analyzed in the discovery phase (n = 58)

| Variables | Spontaneous preterm delivery after sampling | | *P-*value |
| --- | --- | --- | --- |
|  | ≤ 14 days (n = 30) | > 14 days (n = 28) |  |
| Maternal age (years) | 31.8 ± 3.8 | 32.5 ± 3.3 | 0.484**^a^** |
| Nulliparity | 36.6% (11/30) | 46.4% (13/28) | 0.451**^c^** |
| Gestational age at sampling (weeks) | 28.8 ± 1.7 | 27.2 ± 2.8 | **0.035^b^** |
| Gestational age at delivery (weeks) | 29.5± 1.4 | 32.1 ± 2.8 | **<0.001^b^** |
| Sampling-to-delivery interval (days) | 4.5 ± 3.8 | 33.4 ± 16.5 | **<0.001^b^** |
| AF endostatin (ng/mL) | 66.3 ± 25.9 | 63.0 ± 19.4 | 0.975**^b^** |
| AF Fas (ng/mL) | 4.6 ± 1.6 | 4.4 ± 1.9 | 0.692**^a^** |
| AF IL-8 (ng/mL) | 7.5 ± 6.0 | 4.3 ± 5.7 | **0.017^b^** |
| AF lipocalin-2 (µg/mL) | 1.5 ± 0.9 | 0.8 ± 0.9 | **0.004^b^** |
| AF MMP-9 (ng/mL) | 96.7 ± 95.9 | 26.7 ± 55.3 | **0.001^b^** |
| AF S100 A8/A9 (µg/mL) | 28.5 ± 23.7 | 13.4 ± 20.1 | **0.001^b^** |
| Positive AF cultures | 56.6% (17/30) | 35.7% (10/28) | 0.110**^c^** |
| Use of tocolytic agents | 83.3% (25/30) | 57.1% (16/28) | **0.029^c^** |
| Use of antibiotics | 96.6% (29/30) | 96.4% (27/28) | 1.000**^c^** |
| Use of antenatal corticosteroids | 93.3% (28/30) | 89.2% (25/28) | 0.665**^c^** |
| Clinical chorioamnionitis | 20.0% (6/30) | 14.2% (4/28) | 0.732**^c^** |
| Histological chorioamnionitis **^d^** | 66.6% (20/30) | 66.6% (18/27) | 1.000**^c^** |

AF, amniotic fluid; Fas (TNFRSF6), ﬁbroblast-associated **(**tumor necrosis factor receptor superfamily member 6); IL, interleukin; MMP, matrix metalloproteinase; S100A8/A9, S100 calcium binding protein A8/A9 complex.

Data are given as the mean ± standard deviation or % (n/N).

**^a^ Student’s *t*-tests**

**^b^ Mann-Whitney *U*-tests**

**^c^ χ^2^-tests or Fisher’s exact tests,** where **appropriate.**

**^d^** One case was excluded for the analysis because delivery took place at another institution.
