# Supplementary material for: A protein microarray analysis of amniotic fluid proteins for the prediction of spontaneous preterm delivery in women with preterm premature rupture of membranes at 23 to 30 weeks of gestation
Source: PLoS One. 2020 Dec 31;15(12):e0244720. doi: 10.1371/journal.pone.0244720 (PMC7774979; doi:10.1371/journal.pone.0244720)
Supplement: S8 Table — (DOCX) [file pone.0244720.s009.docx]

**S8 Table** Characteristics of the study population grouped by spontaneous preterm delivery within 7 days of sampling in the cohort after excluding the patients analyzed in the discovery phase (n = 58)

| Variables | Spontaneous preterm delivery after sampling | | *P-*value |
| --- | --- | --- | --- |
|  | ≤ 7 days  (n = 23) | > 7 days  (n = 35) |  |
| Maternal age (years) | 32.3 ± 3.0 | 32.1 ± 3.9 | 0.846**^a^** |
| Nulliparity | 34.7% (8/23) | 45.7% (16/35) | 0.408**^c^** |
| Gestational age at sampling (weeks) | 29.4 ± 0.9 | 27.2± 2.7 | **0.001^b^** |
| Gestational age at delivery (weeks) | 29.8 ± 0.9 | 31.3 ± 3.0 | **0.011^a^** |
| Sampling-to-delivery interval (days) | 2.7 ± 2.0 | 28.8 ± 17.5 | **<0.001^b^** |
| AF endostatin (ng/mL) | 68.4 ± 26.5 | 62.3 ± 20.2 | 0.573**^b^** |
| AF Fas (ng/mL) | 4.9 ± 1.7 | 4.4 ± 1.8 | 0.173**^b^** |
| AF IL-8 (ng/mL) | 8.6 ± 5.9 | 4.2 ± 5.5 | **0.002^b^** |
| AF lipocalin-2 (µg/mL) | 1.6 ± 0.9 | 0.8 ± 0.9 | **0.001^b^** |
| AF MMP-9 (ng/mL) | 115.7 ± 96.9 | 29.3 ± 57.8 | **0.003^b^** |
| AF S100 A8/A9 (µg/mL) | 32.5 ± 23.7 | 13.8 ± 19.8 | **0.001^b^** |
| Positive AF cultures | 65.2% (15/23) | 34.2% (12/35) | **0.021^c^** |
| Use of tocolytic agents | 82.6% (19/23) | 62.8% (22/35) | 0.144**^c^** |
| Use of antibiotics | 95.6% (22/23) | 97.1% (34/35) | 1.000**^c^** |
| Use of antenatal corticosteroids | 95.6% (22/23) | 88.5% (31/35) | 0.639**^c^** |
| Clinical chorioamnionitis | 17.3% (4/23) | 17.1% (6/35) | 1.000**^c^** |
| Histological chorioamnionitis^d^ | 69.5% (16/23) | 64.7% (22/34) | 0.703**^c^** |

AF, amniotic fluid; Fas (TNFRSF6), ﬁbroblast-associated **(**tumor necrosis factor receptor superfamily member 6); IL, interleukin; MMP, matrix metalloproteinase; S100A8/A9, S100 calcium binding protein A8/A9 complex.

Data are given as the mean ± standard deviation or % (n/N).

**^a^ Student’s *t*-tests**

**^b^ Mann-Whitney *U*-tests**

**^c^ χ^2^-tests or Fisher’s exact tests,** where **appropriate.**

^d^ One case was excluded for the analysis because delivery took place at another institution.
