# Supplementary material for: A protein microarray analysis of amniotic fluid proteins for the prediction of spontaneous preterm delivery in women with preterm premature rupture of membranes at 23 to 30 weeks of gestation
Source: PLoS One. 2020 Dec 31;15(12):e0244720. doi: 10.1371/journal.pone.0244720 (PMC7774979; doi:10.1371/journal.pone.0244720)
Supplement: S9 Table — Unadjusted and adjusted odds ratios of association between potential amniotic fluid proteins and spontaneous preterm delivery within 14 days in women with preterm premature rupture of membranes in the cohort after excluding the patients analyzed in the discovery phase. (DOCX) [file pone.0244720.s010.docx]

**S9 Table** Multivariable logistic regression model showing the unadjusted and adjusted odds ratios of association between potential amniotic fluid proteins and spontaneous preterm delivery within 14 days in women with preterm premature rupture of membranes in the cohort after excluding the patients analyzed in the discovery phase (n = 58)

| Variables | Odds ratio (95% confidence interval) | | |
| --- | --- | --- | --- |
|  | Unadjusted | Adjusted^a^ | *P*-value^b^ |
| AF IL-8 (ng/mL) | 1.101 (1.002 - 1.210) | 1.093 (0.986 – 1.212) | 0.091 |
| AF lipocalin-2 (µg/mL) | 2.114 (1.169 – 3.824) | 2.426 (1.195 – 4.924) | **0.014** |
| AF MMP-9 (ng/mL) | 1.011(1.004 – 1.019) | 1.011(1.002 – 1.019) | **0.017** |
| AF S100 A8/A9 (µg/mL) | 1.032 (1.006 – 1.059) | 1.028 (1.000 – 1.058) | **0.050** |

AF, amniotic fluid; IL, interleukin; MMP, matrix metalloproteinase; S100A8/A9, S100 calcium binding protein A8/A9 complex.

^a^ For gestational age at sampling and use of tocolytics.

^b^ Of odds ratio adjusted for gestational age at sampling and use of tocolytics.
