# Supplementary material for: A protein microarray analysis of amniotic fluid proteins for the prediction of spontaneous preterm delivery in women with preterm premature rupture of membranes at 23 to 30 weeks of gestation
Source: PLoS One. 2020 Dec 31;15(12):e0244720. doi: 10.1371/journal.pone.0244720 (PMC7774979; doi:10.1371/journal.pone.0244720)
Supplement: S10 Table — Unadjusted and adjusted odds ratios of association between potential amniotic fluid proteins and spontaneous preterm delivery within 7 days in women with preterm premature rupture of membranes in the cohort after excluding the patients analyzed in the discovery phase. (DOCX) [file pone.0244720.s011.docx]

**S10 Table** Multivariable logistic regression model showing the unadjusted and adjusted odds ratios of association between potential amniotic fluid proteins and spontaneous preterm delivery within 7 days in women with preterm premature rupture of membranes in the cohort after excluding the patients analyzed in the discovery phase (n = 58)

| Variables | Odds ratio (95% confidence interval) | | |
| --- | --- | --- | --- |
|  | Unadjusted | Adjusted^a^ | *P*-value^b^ |
| AF IL-8 (ng/mL) | 1.137 (1.032 - 1.253) | 1.154 (1.032 – 1.291) | **0.012** |
| AF lipocalin-2 (µg/mL) | 2.489 (1.363 – 4.546) | 3.422 (1.565 – 7.485) | **0.002** |
| AF MMP-9 (ng/mL) | 1.013 (1.005 – 1.020) | 1.011 (1.003 – 1.020) | **0.006** |
| AF S100 A8/A9 (µg/mL) | 1.038 (1.012 – 1.064) | 1.042 (1.010 – 1.075) | **0.009** |

AF, amniotic fluid; IL, interleukin; MMP, matrix metalloproteinase; S100A8/A9, S100 calcium binding protein A8/A9 complex.

^a^ For gestational age at sampling.

^b^ Of odds ratio adjusted for gestational age at sampling.
