# Supplementary material for: A protein microarray analysis of amniotic fluid proteins for the prediction of spontaneous preterm delivery in women with preterm premature rupture of membranes at 23 to 30 weeks of gestation
Source: PLoS One. 2020 Dec 31;15(12):e0244720. doi: 10.1371/journal.pone.0244720 (PMC7774979; doi:10.1371/journal.pone.0244720)
Supplement: S1 File — (DOCX) [file pone.0244720.s014.docx]

## - Supplemental Materials -

**Methods employed for random selection of cases**

**Following are details on**the **methods employed for random selection of cases: we (1)** created serial numbers **for the list of 45 women with SPTD within 14 days, which was** prepared in a Microsoft Excel **sheet; (2)** generated **a random sequence of numbers between 1 and 45,** using the random sequence generator application; **and (3)** selected 15 cases, **according to the first 15 numbers generated using the** random sequence generator**.**

**Membrane-based human antibody array**

Protein concentrations of each of 30 discovery cohort samples were determined by bicinchoninic acid (BCA) assay (Micro BCA Protein Assay Kit, Thermo Fisher Scientific, Bremen, Germany). The pooled AF samples from imminent SPTD case (iSPTD; n=15) and non-imminent SPTD control (non-iSPTD; n=15) groups were created by combining equal amounts (33.3 μg) of 15 individual AF samples from each group. Consequently, 500 µg of AF samples from each group were mixed and assayed in duplicate, according to the manufacturer’s protocol. The groups’ relative expression levels of immunoregulatory proteins were profiled using a human antibody array kit (AAH-BLM-1B-2; RayBiotech, Norcross, GA), which can simultaneously detect 507 human cytokines and related proteins. The membranes were exposed to a radiographic film (Kodak Industrex Processor Model; Eastman Kodak Company, Rochester, NY, USA), and the signal was detected and quantified using chemiluminescence image analysis (Bio-Rad Quantity 4.6.7; Bio-Rad Laboratories, Inc., Hercules, CA, USA) and the QuantityOne software (Bio-Rad ChemiDoc XRS Systems; Bio-Rad Laboratories, Inc.). The densities were exported to a Microsoft Excel spreadsheet (Microsoft, Redmond, WA, USA). The background intensity was subtracted before analysis, and the signal intensities of each spot were normalized as a percentage of the positive controls on each membrane. To select target proteins showing significant differences in signal intensity between iSPTD and non-iSPTD groups, the following spot selection criteria were applied: fold change of ≥1.5 or ≤0.66 for up- or downregulated proteins upon chemiluminescence image analysis.

**Analysis of various proteins in the amniotic fluid**

The ranges of endostatin, FAS, IL-8, lipocalin-2, MMP-9, S100A8/A9, and S100A10  standard curves were 62.5-4000 pg/mL, 62.5-4000 pg/mL, 31.2-2000 pg/mL, 78.1-5000 pg/mL, 31.2-2000 pg/mL, 93.8-6000 pg/mL, and 0.1-10 ng/ml, respectively. Prior to measurement of these proteins, the amniotic fluid samples were diluted at 1:10 for FAS, IL-8, and MMP-9, 1:100 for endostatin, 1:500 for lipocalin-2, and 1:10000 for S100A8/A9. The intra- and interassay coefficients of variation were 2.8% and 6.3% for endostatin, 6.9% and 6.9% for FAS, 2.6% and 11% for IL-8, 2.6% and 6.5% for lipocalin-2, 7.0% and 13.8% for MMP-9, and 2.2% and 14.5% for S100A8/A9, respectively.

Management of PPROM

Prophylactic broad-spectrum antibiotics (ampicillin plus either azithromycin or erythromycin) were administered in all women with PPROM. Antenatal corticosteroids were administered to mature fetal lungs when PPROM occurred between 24.0 and 34.0 weeks of gestation. Tocolytic therapy (magnesium sulfate, ritodrine, or atosiban) was administered in women with PPROM less than 34 weeks at the discretion of the attending obstetrician. Maternal or fetal health status was carefully monitored for the development of clinical sign of chorioamnionitis and/or fetal compromise, both of which are indications for induction of labor. In most patients with culture-proven microbial invasion of amniotic cavity, labor was not induced or an elective cesarean delivery was not performed purely for positive AF cultures. In women with a diagnosis of intra-amniotic infection (i.e., positive AF cultures) but without clinical chorioamnionitis, the effective antibiotics against isolated bacteria were administered and close monitoring for clinical sign of chorioamnionitis and/or fetal compromise was performed until 34 weeks of gestation. Induction of labor was performed after 34 weeks if clinical sign of chorioamnionitis, fetal compromise or labor had not developed. Acute histologic chorioamnionitis was diagnosed when acute inflammatory change was detected in any tissue sample (umbilical cord, chorionic plate, chorion-decidua, or amnion), in accordance with previously published criteria.^1^ Clinical chorioamnionitis was diagnosed following the criteria proposed by Gibbs et al.^2^; fever (≥37.8°C) and the presence of two or more of the associated clinical findings (uterine tenderness, malodorous vaginal discharge, maternal leukocytosis, maternal tachycardia, and fetal tachycardia).

**Supplementary References**

1. Jung EY, Choi BY, Rhee J, Park J, Cho SH, Park KH. [Relation between amniotic fluid infection or cytokine levels and hearing screen failure in infants at 32 wk gestation or less.](https://www.ncbi.nlm.nih.gov/pubmed/27925622) Pediatr Res. 2017;81(2):349-355.

2. Gibbs RS, Blanco JD, St Clair PJ, Castaneda YS. Quantitative bacteriology of amniotic fluid from women with clinical intraamniotic infection at term. *The Journal of infectious diseases* 1982; 145(1): 1-8.
